# Urinary epidermal growth factor as a prognostic marker for the progression of Alport syndrome in children

**DOI:** 10.1007/s00467-018-3988-1

**Published:** 2018-06-11

**Authors:** Baihong Li, Yanqin Zhang, Fang Wang, Viji Nair, Fangrui Ding, Huijie Xiao, Yong Yao, Matthias Kretzler, Wenjun Ju, Jie Ding

**Affiliations:** 1 Department of Pediatrics, Peking University First Hospital, No.1 Xi An Men Da Jie, Beijing, 100034 People’s Republic of China; 2 Department of Internal Medicine, University of Michigan, Ann Arbor, MI 48109 USA

**Keywords:** Alport syndrome, Urinary epidermal growth factor (uEGF), Progression, Prognostic marker

## Abstract

**Background:**

Alport syndrome is a rare hereditary kidney disease manifested with progressive renal failure. Considerable variation exists in terms of disease progression among patients with Alport syndrome. Identification of patients at high risk of rapid progression remains an unmet need. Urinary epidermal growth factor (uEGF) has been shown to be independently associated with risk of progression to adverse kidney outcome in multiple independent adult chronic kidney disease (CKD) cohorts. In this study, we aim to assess if uEGF is associated with kidney impairment and its prognostic value for children with Alport syndrome.

**Methods:**

One hundred and seventeen pediatric patients with Alport syndrome and 146 healthy children (3–18 years old) were included in this study. uEGF was measured in duplicates in baseline urine samples using ELISA (R&D) and concentration was normalized by urine creatinine (uEGF/Cr). In patients with longitudinal follow-up data (*n* = 38), progression was defined as deteriorated kidney function (CKD stage increase) during follow-up period (follow-up length is about 31 months in average). The association of baseline uEGF/Cr level with estimated glomerular filtration rate (eGFR) slope and Alport syndrome patients’ progression to a more advanced CKD stage during the follow-up period was used to evaluate the prognostic value of the marker.

**Results:**

We found that uEGF/creatinine (uEGF/Cr) decreases with age in pediatric patients with Alport syndrome with a significantly faster rate than in healthy children of the same age group. uEGF/Cr is significantly correlated with eGFR (*r* = 0.75, *p* < 0.001), after adjustment for age. In 38 patients with longitudinal follow-up, we observed a significant correlation between uEGF/Cr and eGFR slope (*r* = 0.58, *p* < 0.001). Patients with lower uEGF/Cr level were at increased risk of progression to a higher CKD stage. uEGF/Cr was able to distinguish progressors from non-progressors with an AUC of 0.88, versus 0.77 by eGFR and 0.81 by 24-h urinary protein (24-h UP).

**Conclusions:**

Our study suggests that uEGF/Cr is a promising biomarker for accelerated kidney function decline in pediatric patients with Alport syndrome. It may help to identify patients at high risk of progression for targeted clinical care and improve the patients’ stratification in interventional trials.

## Introduction

Alport syndrome is a hereditary kidney disease which manifests with hematuria, proteinuria, progressive renal failure, sensorineural deafness, or typical ocular changes, which are caused by mutations in the *COL4A5*, *COL4A3*, or *COL4A4* genes [1]. In current clinical practice, the progression of Alport syndrome is determined by regular measurements of proteinuria and renal function. However, these parameters are not sensitive enough to predict the progression of Alport syndrome. Taking these shortcomings into consideration, additional biomarkers are urgently needed for better prediction of Alport syndrome progression.

Epidural growth factor (EGF) level has been shown to be regulated in kidney diseases [2, 3]. Urinary EGF (uEGF) is reported to be derived from the kidney and has been demonstrated to be downregulated in almost all rodent models of kidney diseases and many kinds of human renal diseases [4–12]. In 2015, Ju et al. reported that the addition of uEGF to creatinine ratio (uEGF/Cr) to standard clinical biomarkers [estimated glomerular filtration rate (eGFR) and urine albumin to creatinine ratio (ACR)] improved the prediction of chronic kidney disease (CKD) progression by following-up three independent diverse CKD cohorts [13]. Furthermore, in 2016, Betz et al. demonstrated that the lower uEGF/Cr at baseline dramatically increased the risk of rapid decline of renal function [14]. These studies further reinforce uEGF/Cr as a potential predictive marker for progressive renal diseases.

The additive value of uEGF/Cr to eGFR and ACR on prediction is likely due to its tissue specificity and the distinct pathogenic pathway it represents. EGF mRNA showed restricted expression in the kidney tubulointerstitium [13]. More specific localization has been reported in the thick ascending limb of Henle and distal tubules [15]. Kidney-restricted origination of uEGF minimized influences from extrarenal tissue/organs therefore can specifically reflect patients’ kidney function. In addition, uEGF is significantly inversely correlated with interstitial fibrosis and tubular atrophy (IF/TA) in patients with nephrotic syndrome [13]. This goes along with its mitogenic function and its reported role in enhancing tubular regeneration and repair [16].

Alport syndrome is well known as a kind of glomerular disease with the typical pathological changes of glomerular basement membrane (GBM) in the last decades. However, in recent years, a lot of studies have described that tubulointerstitial lesions in Alport syndrome, including tubulointerstitial infiltration by CXCR3-positive T cells in human Alport syndrome and interstitial fibrosis, tubule cell apoptosis, and tubular atrophy in animal models with Alport syndrome [17–20]. In addition, treatment of angiotensin-converting enzyme inhibitors (ACEI) in *COL4A3* knockout mice delayed proteinuria and attenuated tubular injury and kidney fibrosis, but has no obvious function for amelioration of pathological changes of GBM [21, 22]. These studies indicated that tubular damage and interstitial fibrosis contribute to the progression of Alport syndrome.

Therefore, we hypothesized that uEGF, a non-invasive biomarker for tubular damage and interstitial fibrosis, can predict progression of Alport syndrome, in which tubulointerstitial impairment is a major contributor of kidney disease progression. In this study, we aimed to determine the prognostic value of uEGF on the progression of kidney impairment in children with Alport syndrome.

## Materials and methods

### Inclusion and exclusion criteria of subjects

#### Healthy children

The inclusion criteria were those (1) under the age of 18 and (2) whose routine urinalysis and urinary albumin creatinine ratio were normal. The exclusion criteria were those (1) with a history of chronic illness; (2) who suffer from infectious diseases (fever, cold, cough, diarrhea, etc.); and (3) have taken medication within the past 1 month.

#### Children with Alport syndrome

The inclusion criteria were those (1) under the age of 18 years old and (2) diagnosed with Alport syndrome by Peking University First Hospital on the basis of the presence of hematuria or a family history of renal diseases with at least one positive examination of GBM changes revealed by electron microscopy, or immunofluorescence of the collagen type IV, or genetic testing of *COL4A3–5*. The exclusion criteria were (1) unconfirmed diagnosis of Alport syndrome and (2) having other kidney diseases besides Alport syndrome.

Alport children included were grouped into four subgroups according to the level of proteinuria and eGFR that is hematuria, microalbuminuria, proteinuria, and renal dysfunction.

- hematuria (24-h urinary protein (24-h UP) < 150 mg; microalbumin < 20 mg/l and eGFR ≥ 90 ml/min/1.73m^2^);
- microalbuminuria (24-h UP < 150 mg; microalbumin ≥ 20 mg/l and eGFR ≥ 90 ml/min/1.73 m^2^);
- proteinuria (24-h UP ≥ 150 mg and eGFR ≥ 90 ml/min/1.73 m^2^);
- renal dysfunction (eGFR < 90 ml/min/1.73 m^2^)

### Urine specimen collection and preparation

Spot urine samples were collected into disposable sterile urine cups from all the subjects included. Urine specimens were centrifuged at 4 °C at 4000 rpm/min for 15 min. The clear supernatants were transferred into new tubes which were immediately stored at − 80 °C. Urine specimens were diluted 200-fold and 250-fold for Alport syndrome and healthy control children respectively with calibrator diluent RD5E before measurements.

### Measurements of uEGF and creatinine

Human EGF Quantikine ELISA kits (DEG00; R&D Systems) were used to measure EGF concentration in human urine. Urine creatinine concentration was measured by a Hitachi Automatic Biochemical Analyzer 7180. Log2 transformed uEGF/creatinine ratio was used for data analysis in this study. Fully validated ELISA assay was used for the measurement of uEGF. Three pre-measured samples with high, medium, or low concentrations were used as quality controls and were included in each plate to determine interplate variation. All samples, quality controls, and standards were run in duplicate. Intra-assay variation is less than 5% and inter-assay coefficients of variation were less than 10%.

#### Definition of Alport syndrome progression

The Alport syndrome progression was defined by two ways: one is using eGFR slope, the rate of eGFR change over the years, as a continuous variable (details in below the “Statistical analysis” section); the other one is CKD stage advance. Progressor was defined as patient whose CKD stage (by Kidney Disease Outcomes Quality Initiative (KDOQI) guideline) is higher at the end of observation than his/her CKD stage at baseline. The equation eGFR = 0.42 × height (cm)/serum creatinine (mg/dl) (ht/scr) was used to calculate the eGFR of children with Alport syndrome [23].

#### Determination of the cutoff of uEGF/Cr value

Alport syndrome children in respective age groups was separated into patients with “lower uEGF/Cr than healthy” and with “normal range uEGF/Cr” based on the cutoff value. In this study, the cutoff value was determined using lower 95% confidence interval (CI) of uEGF/Cr in healthy children in different age groups. Alport syndrome children whose uEGF/Cr were less than the cutoff value for the corresponding age group was defined as “lower uEGF/Cr than healthy,” whereas Alport syndrome children with “normal range uEGF/Cr” referred to those children whose uEGF/Cr concentration is higher than the cutoff value of the corresponding age group.

#### Statistical analysis

Estimated glomerular filtration rate slope determination was described previously [13]. In brief, patients with three or more GFR measurements during the follow-up period were included in fitting a linear mixed effect model GFR_ij = beta0 + beta1 × time_ij + b_0i + b_1i × time_ij + epsilon_ij to calculate eGFR slope percentage change (%) = (beta1 + b_1i) ∕ (beta0 + b_0i). Linear regression was applied to investigate the association between eGFR slope and uEGF/Cr, adjusted for age or baseline eGFR, respectively. Logistic regression and area under the curve (AUC) values from the ROC curves to compare nested models (www.inside-r.org/packages/cran/epicalc/docs/lrtest) were used to evaluate the classification power of the candidate marker. Statistical analysis was performed using R (www.R-project.org) and SPSS software (version 24). To compare skewed variables in multiple independent groups, the Kruskal-Wallis test was used. The Mann-Whitney test was applied to compare skewed distributed variables between two groups.

## Results

### uEGF/Cr decreases with age in healthy children

**Fig. 1:**
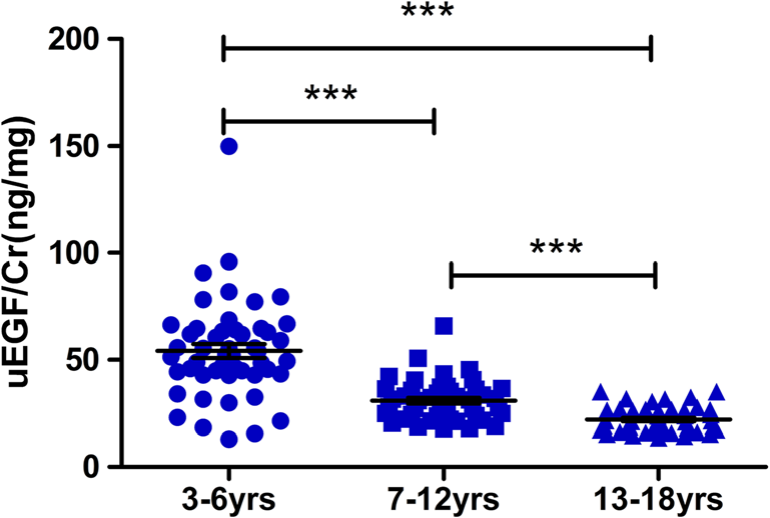
Urinary epidermal growth factor/creatinine (uEGF/Cr) levels in different age group of healthy children. uEGF/Cr of healthy children at 3–6 years (54.17 ± 22.84; *N* = 50) decreased 43% at 7–12 years (30.87 ± 9.37; *N* = 47) and 59% at 13–18 years (22.06 ± 5.78; *N* = 49).Triple asterisks denote *p* < 0.0001

A total of 146 healthy children (3 to18 years old) were included in this study (Table 1). We divided these children into three age groups: pre-school age (3–6 years, *N* = 50), school age (7–12 years, *N* = 47), and adolescence (13–18 years, *N* = 49) and compared the concentration of uEGF/Cr in each group. A significant decrease in uEGF/Cr concentration was observed with increased age (Fig. 1). However, there was no significant difference in uEGF/Cr between males and females in pre-school age (*p* = 0.36), school age (*p* = 0.27), and adolescence (*p* = 0.12) groups, respectively.

**Table 1:** Characteristics of the healthy children and children with Alport syndrome included in this study

Characteristics	All healthy children	All children with Alport syndrome
Number	146	117
Median age (range) (years)	9.5 (3–18)	10 (3–17)
Male (%)	74 (50.7%)	99 (84.6%)
Mean height ± SD (cm)	140.6 ± 25.4	138.4 ± 18.7
Mean weight ± SD (kg)	40.2 ± 21.5	34.7 ± 14.6
Mean 24-h UP ± SD (g/24 h)	NA	1.9 ± 2.3
Mean eGFR ± SD (ml/min per 1.73 m^2^)	NA	110.4 ± 51.1
Genotype (89 of 117)	NA	XLAS (65/89) ARAS (24/89)

*eGFR* estimated glomerular filtration rate, *NA* not available, *UP* urinary protein, *XLAS* X-linked Alport syndrome, *ARAS* autosomal recessive Alport syndrome

### uEGF/Cr is significantly lower in children with Alport syndrome compared to age-matched healthy controls

**Fig. 2:**
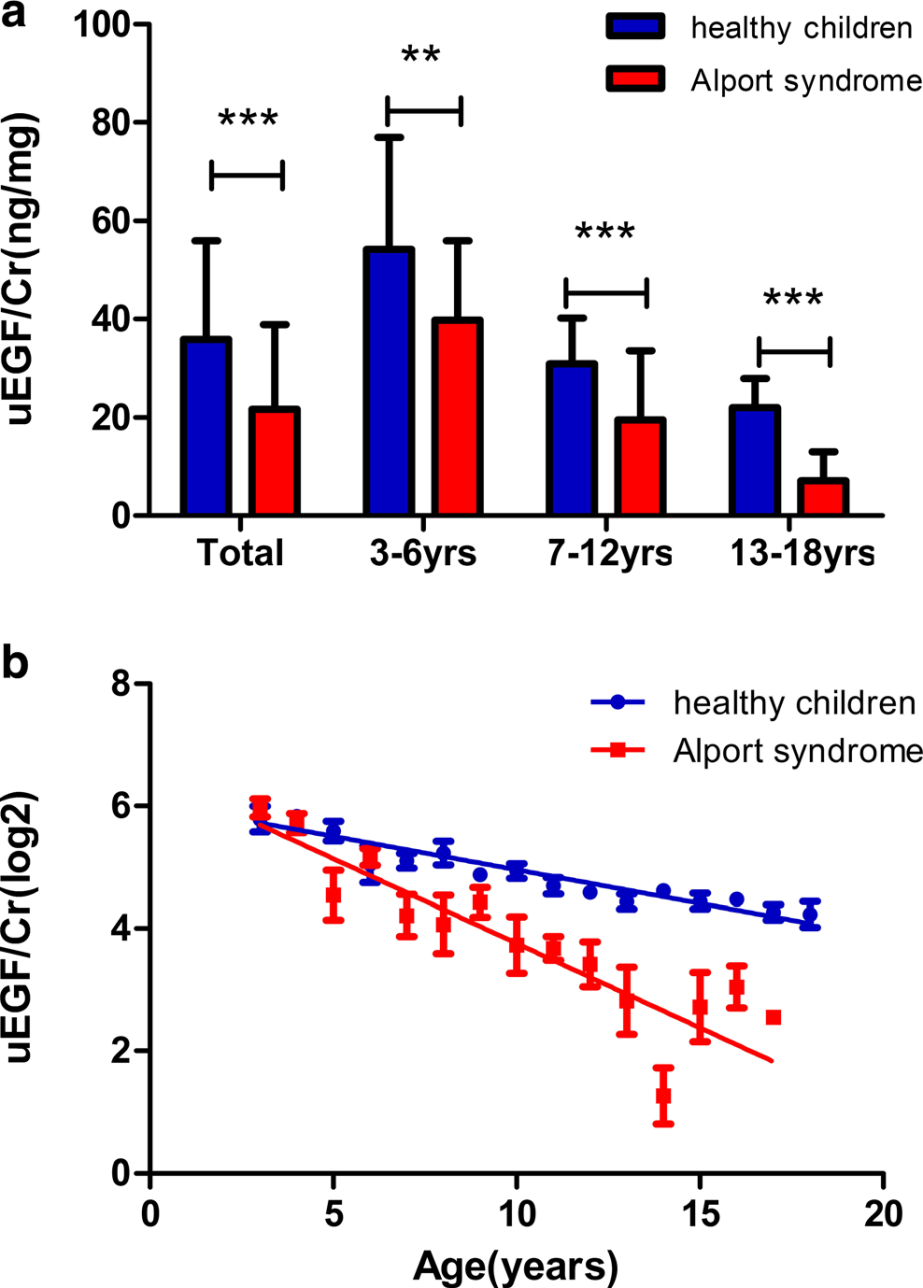
Urinary epidermal growth factor/creatinine (uEGF/Cr) level in different age groups of healthy children and children with Alport syndrome (mean ± SD). **a** uEGF/Cr levels were lower in children with Alport syndrome (red column) in comparison with healthy children (blue column) overall and in each individual age group. Double asterisks represent *p* < 0.01; triple asterisks represent *p* < 0.0001. **b** uEGF/Cr levels decrease with age in healthy children (blue dots, *N* = 146) and children with Alport syndrome (red square, *N* = 117)

A total of 117 Alport children were included in this study (Table 1) from our Alport syndrome registry databank according to the inclusion criteria and exclusion criteria, which are described in detail in the “Materials and methods” section. Comparing the levels of uEGF/Cr in healthy children (*N* = 146) and Alport syndrome children (*N* = 117), the Alport syndrome children showed significantly lower uEGF/Cr compared to healthy children of the same age group. To adjust for age effect, we matched healthy children and Alport syndrome children in all three age groups as described above. Our results showed that uEGF/Cr levels of Alport syndrome children are significantly lower than those of healthy children in all three age groups (Fig. 2a).

Furthermore, uEGF/Cr levels in both Alport syndrome children and healthy children showed a continuous reduction with increased age. uEGF/Cr level in Alport syndrome children, however, fell faster than did the level in healthy children from the same age group (Fig. 2b).

**Fig. 3:**
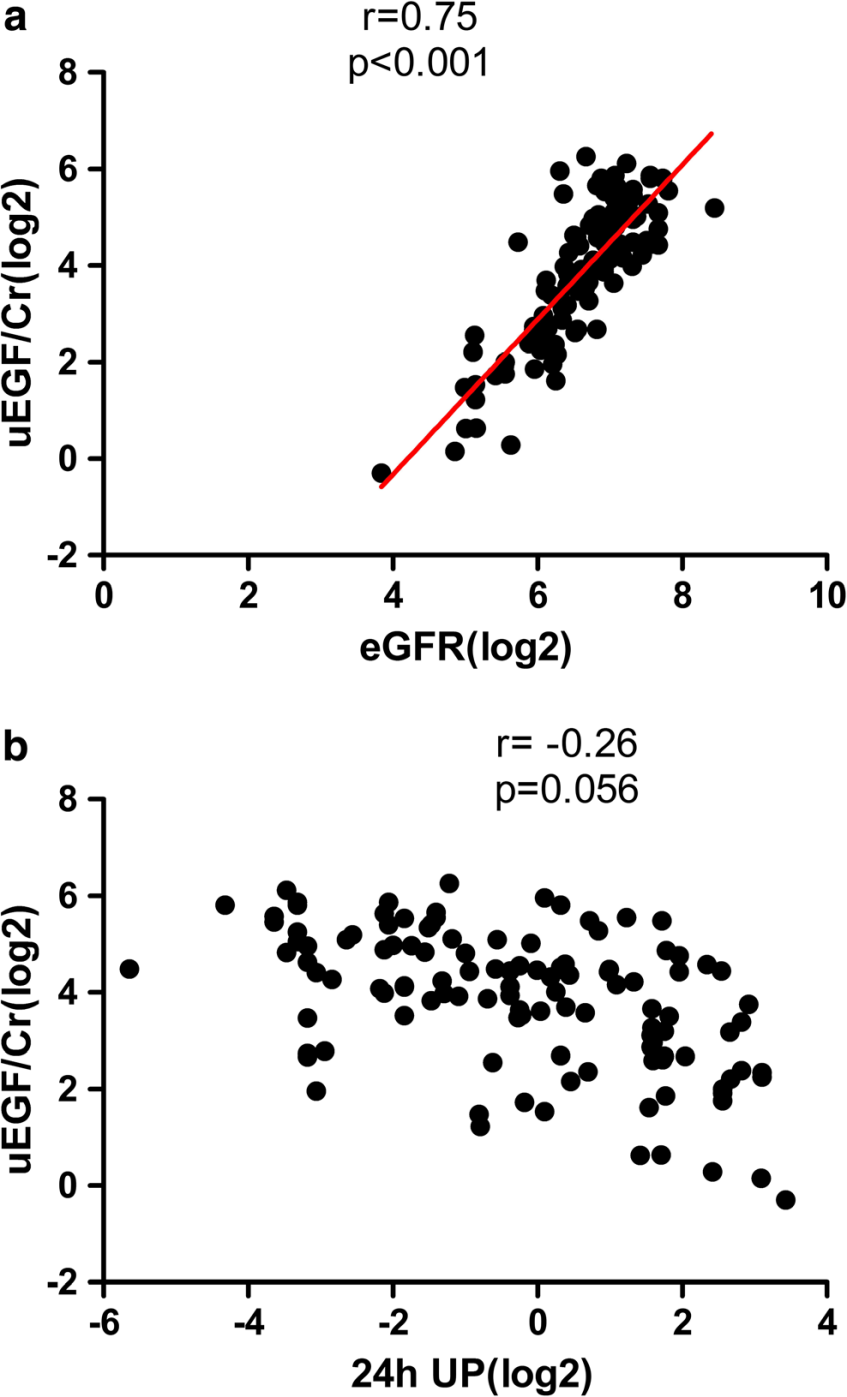
Correlation of one urinary epidermal growth factor/creatinine (uEGF/Cr) with kidney impairment, evaluated by estimated glomerular filtration rate (eGFR) and 24-h UP. **a** uEGF/Cr is significantly (*p* < 0.001) positively correlated with eGFR at the time of urine collection (*N* = 117). **b** uEGF/Cr showed a negative correlation with 24-h UP (*N* = 117), however, did not reach statistical significance

To investigate if uEGF/Cr level is associated with quantitative kidney function in Alport syndrome children, we analyzed the correlation of uEGF/Cr with eGFR and 24-h urinary protein (24-h UP). After adjustment for age, uEGF/Cr showed a tight and significant correlation with eGFR (*r* = 0.75, *p* < 0.001, Fig. 3a). The correlation between uEGF/Cr and 24-h UP is no more significant after adjusting for age (*r* = − 0.26, *p* = 0.06, Fig. 3b).

**Fig. 4:**
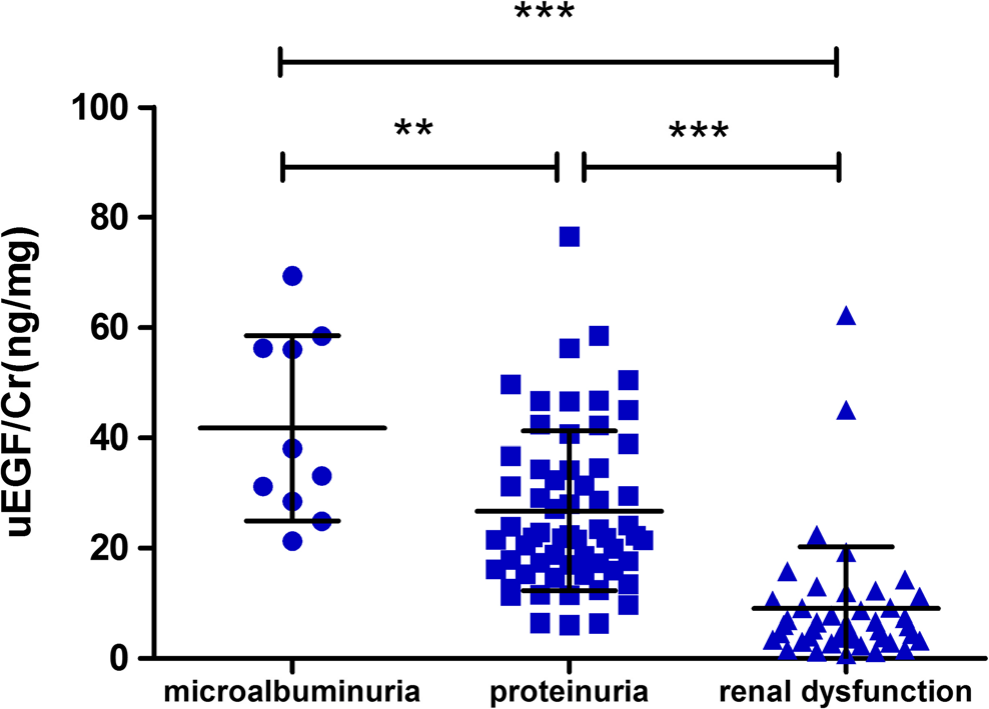
The urinary epidermal growth factor/creatinine (uEGF/Cr) levels (mean ± SD) in children with Alport syndrome. Children were grouped based on their proteinuria levels and estimated glomerular filtration rate (eGFR). Patients with microalbuminuria (*N* = 10), proteinuria (*N* = 61), and renal dysfunction (*N* = 43) were included in this analysis. uEGF/Cr is 36 or 78% less in patients with proteinuria or renal dysfunction in comparison with patients with microalbuminuria, respectively. Double asterisks represent *p* = 0.008; triple asterisks represent *p* < 0.0001

According to the level of proteinuria and eGFR, we divided children with Alport Syndrome into four subgroups, that is, hematuria, microalbuminuria, proteinuria, and renal dysfunction. Patients with proteinuria (24-h UP more than 150 mg but eGFR ≥ 90 ml/min/1.73m^2^) and renal dysfunction (eGFR < 90 ml/min/1.73m^2^) showed a significant reduction of uEGF/Cr compared to Alport children that are only microalbuminuria (Fig. 4). Children with hematuria were not included in this analysis due to the small samples size (*N* = 3) and large variation in this group.

### uEGF/Cr and kidney disease progression in Alport syndrome

In Alport syndrome children with hematuria or microalbuminuria, we observed two distinct subgroups of patients based on uEGF/Cr level. We then asked if different uEGF/Cr levels are associated with future kidney disease progression and can therefore be used as an early prognostic marker.

To answer this question, we investigated those Alport syndrome children whose clinical follow-up data are available. In the 117 Alport syndrome patients, 38 children of school age or adolescence have been followed up for 30.58 ± 16.31 months.

**Fig. 5:**
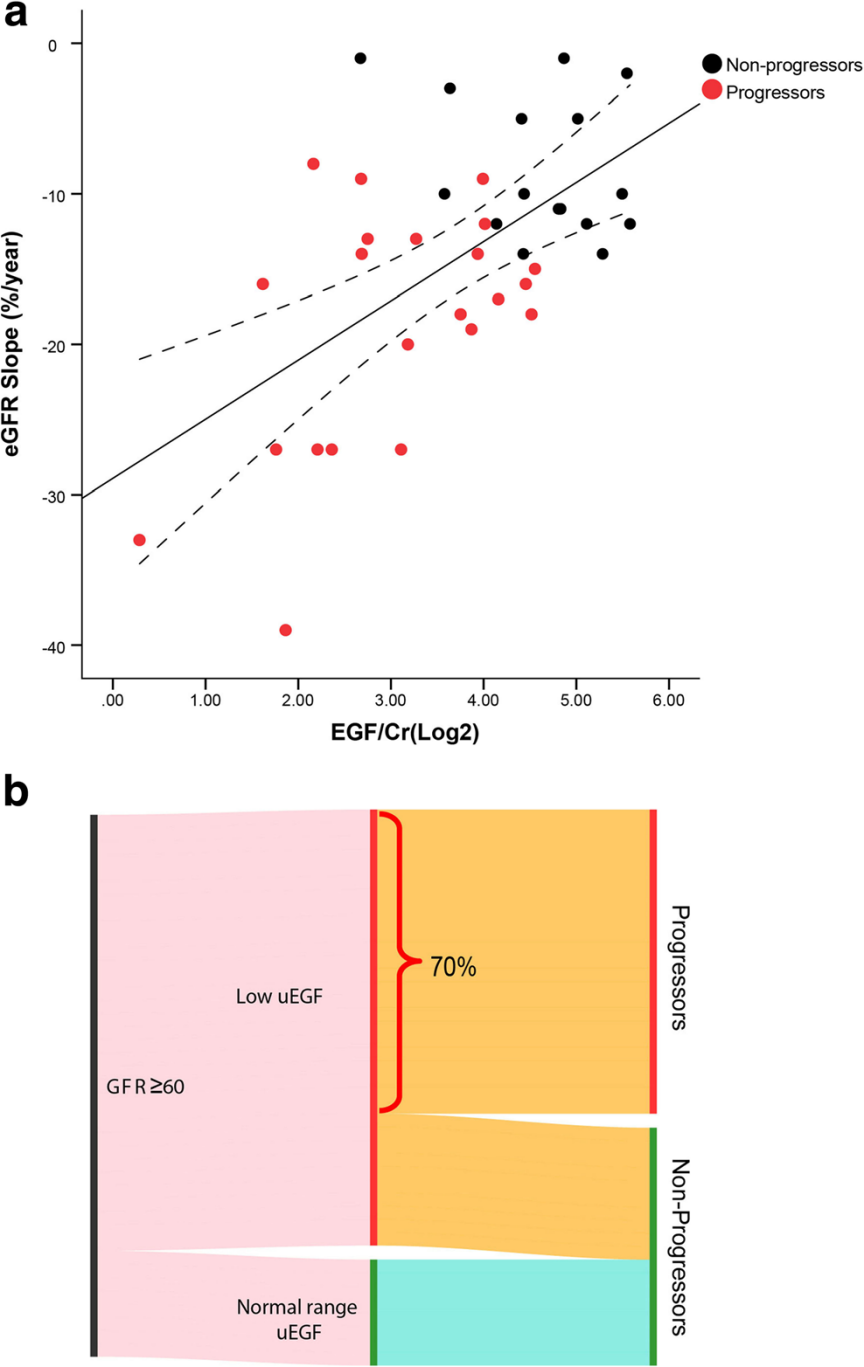
The level of one urinary epidermal growth factor/creatinine (uEGF/Cr) and kidney disease progression in patients with Alport syndrome. **a** uEGF/Cr is significantly and positively correlated with estimated glomerular filtration rate (eGFR) slope (*r* = 0.58, *p* < 0.001). The majority of progressors (red dots) had lower uEGF/Cr and steeper eGFR slope than non-progressors (black dots). **b** Thirty five children with Alport syndrome and eGFR above 60 were divided into “lower uEGF/Cr than healthy” (*N* = 27) and “normal range uEGF/Cr” (*N* = 8) groups based on cutoff values defined based on healthy children of the same age group, as described in the methods section. In patients with eGFR ≥ 60 but lower uEGF/Cr, 70% had progressed to more advanced chronic kidney disease (CKD) stages during follow-up, whereas in patients with normal range uEGF/Cr, none had progressed

Kidney disease progression was defined in two different ways, first by eGFR slope, namely the rate of eGFR change over years. As demonstrated in Fig. 5a, uEGF/Cr is significantly and positively correlated with eGFR slope (*r* = 0.58, *p* < 0.001). The correlation remains significant after adjusting for age (adjusted *r* = 0.46, *p* < 0.01), 24-h UP (adjusted *r* = 0.46, *p* < 0.01), or baseline eGFR (adjusted *r* = 0.33, *p* < 0.05).

Secondly, progression was also defined as patients progressed to a more advanced CKD stage (by KDOQI guideline) during the follow-up period when compared with baseline. “Progressors” referred to patients whose CKD stages at the end of observation were higher than that at baseline. “Non-progressors” were those patients whose CKD stages remained the same or lower than that at baseline. The clinical characteristics of “progressors” and “non-progressors” at baseline and the end of follow-up are depicted in Table 2. Significantly lower baseline uEGF/Cr and eGFR and significantly higher 24-h UP were observed at baseline between “progressors” and “non-progressors.. At the end of follow-up, eGFR remained to be significant whereas 24-h UP was no longer significant.

**Table 2:** Basic clinical characteristics of progressors and non-progressors at baseline and the end of follow-up

	Progressors, *N* = 22	Non-progressors, *N* = 16	*p* value
At baseline			
uEGF/Cr (ng/mg)	10.59 ± 6.83	27.83 ± 12.67	0.0001
Age (years)	11.18 ± 2.63	9.81 ± 2.32	NS
eGFR (ml/min/1.73m^2^)	99.48 ± 37.99	137.80 ± 38.52	0.004
24-h UP (g/24 h)	3.41 ± 2.51^b^	1.31 ± 1.23^b^	0.02
Genotype	XLAS (14/17) ARAS (3/17)	XLAS (11/13) ARAS (2/13)	NS
Baseline CKD stage	CKD1–2 (19/22) CKD3 (3/22)	CKD1–2 (16/16) CKD3 (0/16)	NS
At the end of follow-up			
eGFR (ml/min/1.73m^2^)	59.46 ± 25.63	113.50 ± 20.86	< 0.0001
24-h UP (g/24 h)	4.34 ± 3.78^b^	1.89 ± 2.08^b^	NS
Time (months) ^a^	23.64 ± 13.28	24.75 ± 5.94	NS

Data are shown as mean ± SD; *NS* no significance, *p* > 0.05, *XLAS* X-linked Alport syndrome, *ARAS* autosomal recessive Alport syndrome, *uEGF/Cr* urinary epidermal growth factor/creatinine, *eGFR* estimated glomerular filtration rate, *CKD* chronic kidney disease

^a^“Time” indicates time to stage advance for progressors and time of follow-up for non-progressors

^b^The value for 24-h UP was an average value of *n* = 16 and 14 patients respectively for progressors and non-progressors

To evaluate if males with X-linked Alport syndrome (XLAS) all progressed during the follow-up time, we investigated the 38 Alport patients with longitudinal follow-up. Twenty-three out of 38 patients were males with XLAS and 10 of 23 (43%) had not progressed during the follow-up time. Out of the 23 XLAS male patients, 13/17 (76%) with lower uEGF/Cr progressed and 6/6 (100%) XLAS male patients with normal range uEGF/Cr did not progress. There are only two XLAS female patients; after 2 years’ follow-up, one patient with lower uEGF/Cr progressed and the other one with normal range uEGF/Cr did not progress. Unfortunately, there are only five patients with ARAS and they all have lower uEGF/Cr; three out of five (60%) progressed.

In our study, among the 38 Alport patients with longitudinal follow-up, 30 patients have genetic analysis result available, 12 out of these 30 patients carry missense mutations and 18 carry nonsense mutations, deletion/insertion, or splicing mutations, referred to as “severe mutations.” We investigated the association between different types of mutations and kidney disease progression. We found that 6 out of the 12 patients (50%) in missense mutation group and 11 out of the18 patients (61%) in severe mutation group progressed to advanced CKD stages during the follow-up time. There is no significant difference between missense mutation and severe mutation subgroups (*p* = 0.71) in terms of disease progression.

To determine if patients with lower uEGF/Cr level are more likely to be a progressor, we defined a cutoff value of uEGF/Cr based on the lower 95% CI of uEGF/Cr in healthy children at different age groups to account for age-associated differences (28.12 and 20.40 ng/mg for children at school age and adolescence, respectively) as described in detail in the “Materials and methods” section. Using these cutoff values, 38 Alport syndrome children in respective age groups were divided into two groups: patients with “lower uEGF/Cr than healthy” (*N* = 30) and patients with “normal range uEGF/Cr” (*N* = 8). None of the Alport syndrome children with “normal range uEGF/Cr” progressed, whereas 73% of the Alport syndrome children with “lower uEGF/Cr than healthy” progressed to more advanced CKD stage at the end of follow-up compared to their baseline stage (Fig. 5b). Figure 5b demonstrated that for patients whose eGFR are over 60, uEGF/Cr can help to further stratify the patients: no progression has been observed in patients with normal range uEGF/Cr, whereas if patients have lower than healthy uEGF/Cr, despite the current “preserved” renal function, 70% of the patients (whose eGFR are over 60 but has lower uEGF/Cr) have proceeded to a more advanced stage in about 2 years of time.

Finally, the association of baseline uEGF/Cr with progression to more advanced stage was evaluated by classification power [comparing the area under receiver operating characteristic (ROC) curves of individual variables and the corresponding models] and survival analysis (time-to-event analysis). ROC analysis showed uEGF/Cr exhibits the highest area under the curve (AUC) of 0.88 (95% CI, 0.77 to 0.99), compared to 24-h UP [0.81 (0.67 to 0.95)] and eGFR [0.77 (0.62 to 0.92)]. The AUC for the base model (including eGFR and 24-h UP, adjusted for age and gender) is 0.86 (0.74 to 0.97); addition of uEGF/Cr to the base model increased AUC to 0.93 (0.85 to 0.99). Addition of uEGF/Cr resulted in a statistically improved model as evaluated by the likelihood test (*p* = 0.002).

## Discussion

Patients with Alport syndrome show a large variation in rate of progression [24–26]. Genetic test may detect the causal mutations, however, cannot accurately predict if when and how fast the patients progress. As shown in our study, not all males with X-linked Alport syndrome progressed during the follow-up time. Due to lack of sensitive and specific prognostic biomarkers, patients at high risk of progression often miss the best time for treatment. To address this urgent need, we investigated if urinary EGF is associated with loss of kidney function and if it has prognostic value in patients with Alport syndrome, since urinary EGF has been reported previously as a prognostic marker in adult CKD progression. Our data showed that in 117 patients with a definitive diagnosis of Alport syndrome and no other kidney diseases, uEGF is highly and significantly correlated with baseline eGFR but not with 24-h proteinuria. These findings are consistent with previous findings in general CKD, IgAN, and nephrotic syndrome [13].

In a subgroup of patients whose follow-up data were available, we attempted to investigate the association of uEGF/Cr with progression of Alport syndrome. Due to small sample size and relatively short follow-up time, we defined “progression” as CKD stage advanced. This definition has been applied in multiple studies by independent groups and was considered acceptable [27–30]. We observed that uEGF/Cr levels at baseline in “progressors” were already significantly lower than those in “non-progressors.” Next, uEGF/Cr’s prognostic value was reflected by its significant correlation with eGFR slope (Fig. 5a). The progressors were clustered together by their low uEGF/Cr and steeper eGFR slope (Fig. 5a). While it is not surprising that steeper eGFR slope highly associated with progression to higher CKD stage, it is encouraging to observe that uEGF/Cr shows a high correlation with eGFR slope and progression. This result confirmed our previous finding in general adult CKD [13]. The association of uEGF/Cr with progression is likely due to the regenerative function of EGF [16]. Lower uEGF/Cr is not only an indication of current reduced kidney tubular mass thereby a good biomarker for cross-sectional kidney function, it also represents impaired regenerative function of the tubular cells, which is directly associated with progression. Finally, uEGF/Cr exhibited superior AUC compared to eGFR and proteinuria. Adding uEGF/Cr to the base model including eGFR and proteinuria resulted in a significant improvement of AUC. This is the first time, to the best of our knowledge, uEGF/Cr was reported to have additive prognostic value for CKD progression in patients with Alport syndrome.

A good biomarker represents the active mechanism underlying the pathogenesis of the disease. Alport syndrome is a well-known glomerular disease with typical pathological changes of the glomerular basement membrane. However, tubulointerstitial fibrosis, a hallmarker for kidney disease progression, has been reported in patients with Alport syndrome [21, 31]. IF/TA has been reported to be well represented by uEGF, a biomarker for tubulointerstitial damage [13]. This could explain the additive value of uEGF/Cr to eGFR and proteinuria, as the two standard clinical markers mainly reflect glomerular damage. If and how EGF is actively involved in Alport syndrome tubular damage repair and regeneration in Alport syndrome remains to be further studied.

It has been reported that there is a consistent downward trend of uEGF/Cr as a function of age from birth to 16 and from 20 to 79, respectively [32, 33]. Therefore, all analyses in our study have been adjusted for age as a significant confounding factor. Inevitably, the uEGF/Cr concentration in Alport syndrome children is an integrated outcome of the normal growth of the kidney and the kidney disease influence. Therefore, there is a need to evaluate age-associated uEGF/Cr reduction in healthy children to allow better correction of the confounding effect. We therefore started establishing a cohort of healthy children aged 3 to 18 years old. Using these healthy children as reference, we could demonstrate that uEGF/Cr levels of Alport syndrome children exhibited a much steeper trajectory over age compared to the reduction in healthy kids of the same age group (Fig. 2b).

The relevant next step would be to define an age-dependent normal range of uEGF/Cr. Once established, it will allow us to identify children with preserved renal function but may have lower than normal uEGF/Cr. As illustrated in Fig. 5b, 70% of Alport syndrome patients whose eGFR is above 60 but have lower uEGF/Cr (compared to healthy kids) progressed to a more advanced CKD stage in an average of about 30 months of follow-up time. Therefore, uEGF/Cr may contribute to the early identification of patients at high risk of kidney disease progression.

Our study has limitations. The small sample size (due to the rare disease nature) and short follow-up restricted us from performing standard time-to-event analysis using FDA approved kidney disease endpoint definition. Despite the small sample size, there were six patients who progressed to end-stage renal disease (ESRD) during the follow-up period. We evaluated the association of uEGF/Cr with time to ESRD using univariate Cox regression model and found that lower uEGF/Cr was significantly associated with increased risk of progression to ESRD (HR = 0.54, *p* = 0.001). However, due to the small event size, no multivariable model can be reliably applied; therefore, it remains to be studied if the association with time to ESRD is independent of eGFR or proteinuria. This is certainly worth further investigation in well-designed clinical studies with larger patient population. In addition, the clinical phenotype and outcomes of individuals with Alport syndrome (especially females with COL4A5 mutation, heterozygous autosomal mutation) are heterogeneous. It has been reported that 12% of females with COL4A5 mutation developed ESRD before the age of 40 years and there is no genotype-phenotype associations in the studied cohort [34]. Similar results have been reported by Yamamura et al., who analyzed female patients with X-linked Alport syndrome from 179 Japanese families and found that 15% of patients reached ESRD by the age of 40 [35]. Patients with autosomal-dominant Alport syndrome had a wide range of phenotypes from ESRD to isolated microhematuria [36, 37]. Therefore, identification of individuals with Alport syndrome, especially females with COL4A5 mutation and heterozygous autosomal mutation at high risk of rapid progression is indeed necessary for early and targeted treatment. From our current results, uEGF is likely a prognostic marker for risk evaluation in this population, which warrants further investigation in large study cohort.

Taken together, our data indicated that Alport syndrome children with a lower expression of uEGF/Cr were at higher risk of rapid progression, even if their eGFRs are still above 60. This finding supports the use of uEGF/Cr to improve early identification of patients at high risk of progression. In addition, this study provided additional data on uEGF/Cr in healthy children from age 3 to 18, contributed to the understanding of the expression of uEGF/Cr as a function of increasing age in healthy people. Prospective study includes more patients (particularly those who have low uEGF/Cr) and longer follow-up is required to validate the prognostic value of uEGF/Cr in children with Alport syndrome.

## Abbreviations

24-h UP: 24-h urinary protein
ACEI: Angiotensin-converting enzyme inhibitors
ARAS: Autosomal recessive Alport syndrome
CI: Confidence interval
eGFR: Estimated glomerular filtration rate
ESRD: End-stage renal disease
GBM: Glomerular basement membrane
IF/TA: Interstitial fibrosis/tubular atrophy
ROC: Receiver operating characteristic
uEGF: Urinary epidermal growth factor
uEGF/Cr: Urinary epidermal growth factor/creatinine
XLAS: X-linked Alport syndrome
